# Serum repressing efflux pump *CDR1 *in *Candida albicans*

**DOI:** 10.1186/1471-2199-7-22

**Published:** 2006-07-13

**Authors:** Yun-Liang Yang, Yi-Hsuan Lin, Ming-Yang Tsao, Chia-Geun Chen, Hsin-I Shih, Jen-Chung Fan, Jang-Shiun Wang, Hsiu-Jung Lo

**Affiliations:** 1 Department of Biological Science and Technology, National Chiao Tung University, Hsinchu; 2 Division of Clinical Research, National Health Research Institutes, Miaoli; 3 Graduate Institute of Life Sciences, National Defense Medical Center, Taipei, Taiwan, Republic of China

## Abstract

**Background:**

In the past decades, the prevalence of candidemia has increased significantly and drug resistance has also become a pressing problem.  Overexpression of *CDR1*, an efflux pump, has been proposed as a major mechanism contributing to the drug resistance in *Candida albicans*. It has been demonstrated that biological fluids such as human serum can have profound effects on antifungal pharmacodynamics. The aim of this study is to understand the effects of serum in drug susceptibility via monitoring the activity of *CDR1 *promoter of *C. albicans*.

**Results:**

The wild-type *C. albicans *cells (SC5314) but not the *cdr1/cdr1 *mutant cells became more susceptible to the antifungal drug when the medium contained serum. To understand the regulation of *CDR1 *in the presence of serum, we have constructed *CDR1 *promoter-Renilla luciferase (*CDR1p-RLUC*) reporter to monitor the activity of the *CDR1 *promoter in *C. albicans*. As expected, the expression of *CDR1p-RLUC *was induced by miconazole. Surprisingly, it was repressed by serum. Consistently, the level of *CDR1 *mRNA was also reduced in the presence of serum but not N-acetyl-D-glucosamine, a known inducer for germ tube formation.

**Conclusion:**

Our finding that the expression of *CDR1 *is repressed by serum raises the question as to how does *CDR1 *contribute to the drug resistance in *C. albicans *causing candidemia. This also suggests that it is important to re-assess the prediction of *in vivo *therapeutic outcome of candidemia based on the results of standard *in vitro *antifungal susceptibility testing, conducted in the absence of serum.

## Background

In the past decades, the prevalence of candidemia has increased significantly. Among them, *Candida albicans *is the most frequently isolated fungal pathogen in humans and has caused morbidity in seriously debilitated and immunocompromised hosts. Coinciding with the increased usage of antifungal drugs, the incidences of drug resistance have also increased [1,2].

Overexpression of *CDR1*, an ATP binding cassette (ABC) transporter, has been shown to be the major mechanism for the drug resistance of clinical isolates [3]. Mutations on *CDR1 *in *C. albicans *have resulted in an increased susceptibility to azole drugs [4], which is consistent with the observation that overexpression of *CDR1 *contributes to the drug resistance of clinical isolates of *C. albicans *[5]. Interestingly, the expression of *CDR1 *is increased approximately 4-fold in *Catup1/Catup1 *mutant cells, which are predominately in the hyphal form [6]. This data suggests that CaTup1 acts as a negative regulator of *CDR1*. Recently, two transcription factors, *CaNDT80 *and *CaTAC1*, have been identified as positive regulators of *CDR1 *in *C. albicans *[7,8].

Previous works have demonstrated that biological fluids such as human serum can have profound effects on antifungal pharmacodynamics [9]. During an infection, the *C. albicans *cells exist in the host body and are surrounded by blood and other body fluid, where they encounter the antifungal drugs. In this study, we have found that the wild-type SC5314 cells but not the *cdr1/cdr1 *mutant cells became more susceptible to fluconazole, a commonly used antifungal drug, when the medium contained serum. To investigate the regulation of *CDR1 *in the presence of serum, we have constructed a *CDR1 *promoter-Renilla luciferase gene (*CDR1p-RLUC*) reporter to monitor the activity of *CDR1 *promoter in *C. albicans *under different conditions. In conclusion, serum increases the drug susceptibility by repressing the expression of *CDR1*.

## Results and discussion

**Figure 1:**
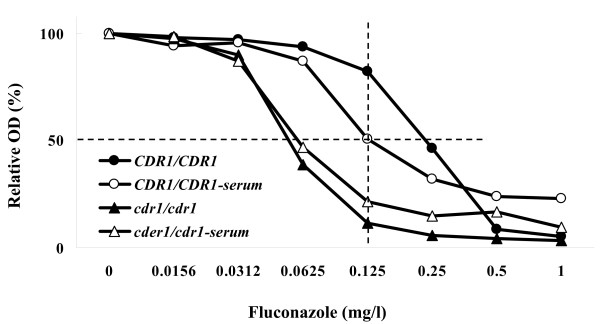
**Fluconazole susceptibility testing**. The inhibition on growth by fluconazole was determined by microdilution method. The wild-type strain (SC5314, squares) and the *cdr1/cdr1 *mutant cells (DSY448, triangles) were grown in the absence (solid symbols) or presence of 10% fetal bovine serum (open symbols). The optical density (OD) of each well was read at 600 nm after incubated at 35°C for 48 hours. The horizontal dotted line represents 50% inhibition and the vertical dotted line refers the inhibition by 0.125 mg/l fluconazole.

To determine if serum has any effect on the drug susceptibility of *C. albicans*, we have determined the growth of cells in the presence of different concentrations of fluconazole and in the absence or presence of 10% fetal bovine serum. The wild-type cells but not the *cdr1/cdr1 *mutant cells became more susceptible to fluconazole when the medium contained serum (Fig. 1). The growth of the wild-type cells was less inhibited by 0.125 mg/l fluconazole in serum-free medium than that in serum-containing one (vertical dotted line). The minimum inhibitory concentration (MIC) of fluconazole of the *cdr1/cdr1 *mutant cells (0.0625 mg/l) was lower than that of the wild-type cells (0.25 mg/l). This is consistent with the previous reports that mutations on *CDR1 *have resulted in an increased drug susceptibility [4,7]. The MIC of fluconazole of the wild-type cells was 2-fold lower in serum-containing medium than that in serum-free one (0.125 mg/l vs. 0.25 mg/l).

**Figure 2:**
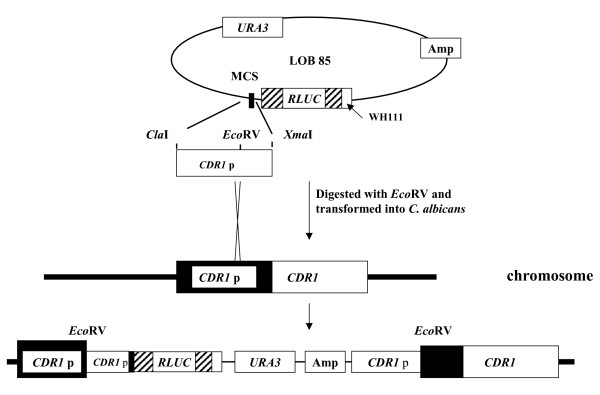
**Construction of *CDR1 *promoter-Renilla luciferase**. The PCR products containing the promoter and the translation initiation site of *CDR1 *(open box) were introduced in-frame with the sequence encoding luciferase (*RLUC*, hatched box) into the plasmid pGEM with the wild-type *URA3 *gene as the selective marker. The LOB 85 plasmid was linearized with *Eco*RV, transformed, and integrated into the chromosome at the promoter of *CDR1 *(solid box) in *C. albicans*.

**Figure 3:**
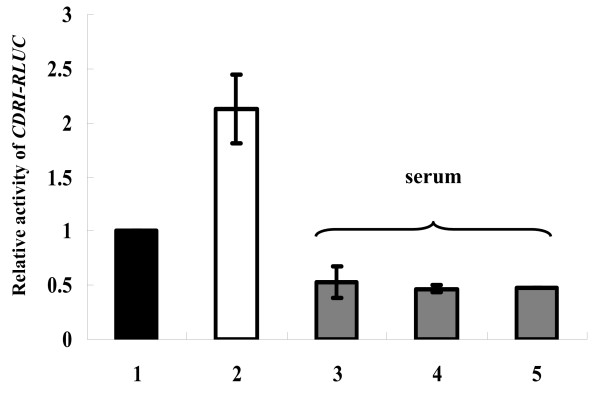
**The activity assay of *CDR1 *promoter-Renilla luciferase gene**. The activity of luciferase from control cells (bar 1) was defined as one. The activity of luciferase from cells in the presence of miconazole or sera was normalized accordingly. Bar 1, with neither serum nor miconazole; bar 2, with 100 μg/ml of miconazole; bar 3, with 10% fetal bovine serum; bars 4 and 5, with 10% human serum.

It is generally accepted that overexpression of *CDR1 *is the major mechanism for the drug resistance of clinical isolates [3,5,10]. Our finding that the wild-type cells but not the *cdr1/cdr1 *mutants became more susceptible to fluconazole in serum-containing medium led us to further investigate the effects of serum on *CDR1 *expression. First of all, we have constructed a *CDR1p-RLUC *reporter (Fig. 2). The construct was integrated into the *CDR1 *locus and its activities under different conditions in different strains were determined. The results are summarized in Figure 3. The *C. albicans *cells containing *CDR1p-RLUC *were harvested after being grown in Synthetic Dextrose (SD) liquid medium in the absence or presence of 100 mg/l of miconazole at 35°C for one hour. The expression of *CDR1p-RLUC *was induced approximately 2-fold by miconazole (Fig. 3, comparing bar 1 to bar 2). This datum is consistent with the previously report that the expression of *CDR1 *is induced by miconazole [11] and also suggests that the activity of *CDR1p-RLUC *can refer the expression of *CDR1 *under our experimental setup.

To determine the effect of serum on the expression of *CDR1*, we have measured the activities of *CDR1p-RLUC *in *C. albicans *cells that were grown in SD liquid medium in the absence or presence of 10% fetal bovine serum at 35°C for one hour. Surprisingly, the serum repressed the expression of *CDR1p-RLUC*. In the presence of the serum, the expression of *CDR1p-RLUC *was reduced to 50% of that in the absence of the serum (Fig. 3, comparing bar 1 to bar 3). To determine if human serum also has the same effect, we have also cultured the cells in the presence of 10% human serum from two healthy volunteers. Interestingly, like the fetal bovine serum, the human sera also reduced the expression of *CDR1p-RLUC *(Fig. 3, comparing bar 1 to bars 4 and 5).

**Figure 4:**
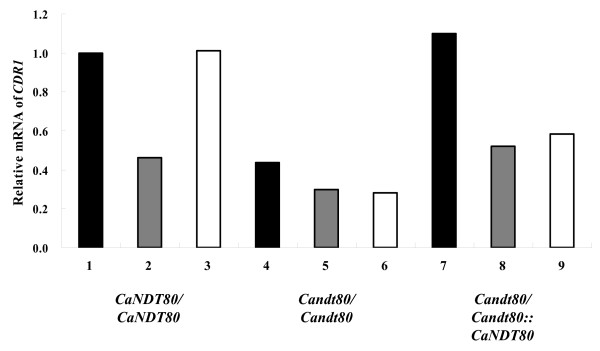
**The level of *CDR1 *mRNA**. Total RNA was isolated from cells in the absence of serum (bars 1, 4, 7), in the presence of 10% fetal bovine serum (bars 2, 5, 8), or in the presence of 5 mM Glu-NAc (bars 3, 6, 9). The level of RNA isolated from the wild-type cells in the absence of serum was defined as one and the relative level of mRNA isolated from different strains was normalized accordingly. Bars 1–3, *CaNDT80/CaNDT80 *(SC5314); bars 4–6, *Candt80/Candt80 *(YLO133), bars 7–9, *Candt80/Candt80::CANDT80 *(YLO137).

This datum suggests that if the activity of *CDR1p-RLUC *echoes the expression of endogenous *CDR1*, the level of *CDR1 *mRNA would be reduced when *C. albicans *cells were grown in the presence of serum. To investigate this hypothesis, we have measured the level of *CDR1 *mRNAs in different strains by real-time PCR. In the presence of 10% fetal bovine serum, the level of *CDR1 *mRNA in the wild-type cells was reduced to approximately 50% of that in the absence of the serum (Fig. 4, comparing bar 1 to bar 2), which re-assured the result of the activity assay with *CDR1p-RLUC*. These results conclude that serum represses the expression of *CDR1*. Since serum is a key stimulant for hyphal formation of *C. albicans*, we would like to determine whether its effect on *CDR1 *expression is due to a factor solely present in serum or common in other hypha-inducing media also. Consequently, we have determined if 5 mM N-acetyl-D-glucosamine (Glu-NAc), a known inducer for germ tube formation of *C. albicans *would alter the expression of *CDR1 *[12,13]. Unlike serum, Glu-NAc did not have effect on the expression of *CDR1 *(Fig. 4, comparing bar 1 and bar 3). This result suggests that the expression of *CDR1 *is not repressed by every hypha-inducing medium. This is consistent with the previously reported that the expression of *CDR1 *is increased in *Catup1/Catup1 *mutant cells, which are predominately in hyphal form [6].

Recently, CaNdt80 has been identified as a positive regulator of *CDR1 *in *C. albicans *[7]. To investigate if serum represses the expression of *CDR1 *via CaNdt80, we have also determined the level of *CDR1 *in *Candt80/Candt80 *mutant cells. The expression of *CDR1 *was reduced 50% by the null mutation of *CaNDT80 *(Fig. 4, comparing bar 1 to bar 4), which is consistent with our previous finding that CaNdt80 regulates *CDR1 *positively [7]. If regulating the expression of *CDR1 *by serum is independent of the activity of CaNdt80, the level of *CDR1 *mRNA in the *Candt80/Candt80 *mutant cells would be significantly reduced in the presence of serum. Otherwise, it will not (if there is any). Our data showed that although 10% fetal bovine serum further reduced the expression of *CDR1 *in the *Candt80/Candt80 *mutant cells, the effect was mild (Fig. 4, comparing bar 4 to bar 5).

## Conclusion

We have found that the wild-type cells became more susceptible to fluconazole when the medium contained serum. Furthermore, the expression of *CDR1 *is repressed significantly by serum according to the activity of the reporter and *CDR1 *mRNA level. The level of *CDR1 *mRNA was only mildly reduced by serum in the *Candt80/Candt80 *mutant cells suggesting the major, if not the sole, regulatory ability of serum may be through the activity of CaNdt80. However, we still can not rule out the possibility that serum may also act through Tac1 and/or other unidentified regulators. It will be interesting to investigate the coordination between CaNdt80 and Tac1 in regulating the expression of *CDR1 *in the presence of serum.

The standard antifungal susceptibility testing [14], which is conducted in the absence of serum, is unreliable in predicting the clinical outcome of therapies, especially for systemic infections. Our finding may explain the existence and persistence of such a discrepancy between the susceptibilities of *in vivo *and *in vitro *environments.

## Methods

### Strains and media

Strains of *C. albicans *used in this studyare as following: SC5314, the wild-type control strain; DSY448, *ura3*Δ:: λ*imm434*/*ura3*Δ:: λ*imm434*; DSY448, *ura3*Δ:: λ *imm434*/*ura3*Δ:: λ*imm434 cdr1::hisG/cdr1::hisG-URA3-hisG*, a gift from Dr. D. Sanglard [4]; YLO133, *ura3*Δ:: λ*imm434*/*ura3*Δ:: λ*imm434 his1::hisG/his1::hisG arg4::hisG/arg4::hisG Candt80::GFP-Arg4/Candt80::URA3-dpl20 ENO1/eno1::ENO1-tetR-SCHAP4-3xHA-HIS1*; and YLO137, *ura3*Δ:: λ*imm434*/*ura3*Δ:: λ*imm434 his1::hisG/his1::hisG arg4::hisG/arg4::hisG Candt80::GFP-Arg4/Candt80:: URA3-dpl200:: CaNDT80::HIS1 *[7]. Yeast Peptone Dextrose (YPD) contained 1% yeast extract, 2% peptone, and 2% dextrose and Synthetic Dextrose (SD) contained 0.67% yeast nitrogen base without amino acid and 2% dextrose. All agar plates were prepared with addition of 2% agar in media.

### Antifungal drug susceptibility

The minimum inhibitory concentration (MIC) to fluconazole of each strain was determined by *in vitro *antifungal susceptibility testing using microdilution method according to published guidelines by the Clinical and Laboratory Standards Institute (CLSI, formerly NCCLS) [14]. The antifungal agent fluconazole (Pfizer, Inc.) was freshly prepared as a stock at the concentration of 16 g/l in DMSO. For working concentration, (16~0.0312 μg/ml), it was processed by stepwise twofold dilutions in SD medium. A drug-free culture and a sterile control were included in each microtitre plate. The optical density (OD) in each well of the microtitre plate was read with a microplate reader (Molecular Devices, SPECTRA MAX plus) at 600 nm after incubated at 35°C for 48 hours. The drug inhibitory curve was presented by the OD of each well with different concentrations of fluconazole relative to the OD of the drug-free control. The MIC was defined as the lowest concentration which reduced the culture broth turbidity by 50%.

### Construction of *CDR1* promoter-Renilla luciferase

A 1.2 kilo-base-pair (kb) DNA fragment containing the *RLUC *gene modified for *C. albicans *with the *WH11 *transcription termination sequence at the 3' end of the *RLUC *open reading frame was isolated from pCRW3 [11] after digested with *Eco*RV and *Nco*I. The purified DNA fragment was blunt-ended with klenow and then ligated to the pGEM-*URA *[15] at the *Nae*I site to construct the plasmid LOB60. Another 1.2 kb DNA fragment containing the promoter and the translation initiation site of *CDR1 *was generated by using oligonucleotides HJL340, 5'-d(GATCATCGATACTCAATAAG) and HJL 341, 5'-d(CGCAAGCCCGGGTAATTTTTTTC). The plasmid LOB60 was then used to construct plasmid LOB85 by introducing the PCR product at restriction sites of *Cla*I and *Xma*I (Fig. 2). The resulted LOB85 was then linearized with *Eco*RV at the 455 base-pair (bp) upstream of the translation initiation site of *CDR1 *and used for transformation to integrate into the chromosome at the promoter of *CDR1 *of CAI4 to produce the Ura3^+ ^transformant, YLO185, *ura3*Δ:: λ*imm434*/*ura3*Δ:: λ*imm434 CDR1p-RLUC-URA3 *(Fig. 2).

### Activity assay of *CDR1* promoter-Renilla luciferase gene (*CDR1p-RLUC*)

Overnight pre-cultured *C. albicans *cells containing *CDR1p-RLUC *were diluted in 10 ml of the SD liquid medium to a final concentration about 4 × 10^6 ^cells per ml. Prior to the addition of serum or miconazole, the dilutents were incubated at 30°C for 4 hours. The cells were harvested after treated with 10% serum (either the fetal bovine serum provided by JRH Biosciences, Australia or the human serum from two coauthors) or 100 μg/ml of miconazole (Sigma M-3512) at 35°C for one hour. The control cells were grown in SD medium in the absence of serum and miconazole at 35°C for one hour. The cells were resuspended in lysis buffer for luciferase assay using the Dual-Glo Luciferase Assay System (E2940, Promega, Madison, USA). The activity of luciferase was determined according to the protocol provided by the manufacturer. The activity of luciferase in the control cells without treatment was defined as one and the relative activity of luciferase in cells with other treatments was normalized accordingly.

### Quantitative analysis of the mRNA level by Real-Time PCR

The *C. albicans *cells were harvested at an OD_600 _between 0.7 and 0.9 after being grown in 20 ml of the SD liquid medium in the absence or presence of 10% fetal bovine serum (JRH Biosciences, Australia) or 5 mM Glu-NAc (Sigma, A8625) at 37°C for one hour. A real-time PCR was performed in a Rotor-Gene™ 3000 instrument (Corbett Research, Australia) with a TITANIUM™ Taq PCR kit (BD Clontech 639210) and SYBR^®^Green I Nucleic Acid Stain (Cambrex 50513) to determine the level of mRNA. The sample was automatically setup by CAS-1200™ (Corbett Research, Australia). The real-time PCR was performed according to the instructions from the manufacturer. The expression of *TEF3 *in each strain was used as the control. The relative quantitation was based on two standard curves for comparisons and the results were given as a ratio [16]. The level of *CDR1 *mRNA isolated from the wild-type cells in the absence of serum was defined as one. The relative level of mRNA isolated from different strains was normalized accordingly.

## Authors' contributions

YLY designed the study and drafted the manuscript with contribution from HJL. MYT and HIS constructed *CDR1p-RLUC *and YHL, CGC, JCF and JSW performed experiments.
